# Hyperhomocysteinemia Presenting as Stroke in a Young Individual: A Case Report

**DOI:** 10.7759/cureus.52381

**Published:** 2024-01-16

**Authors:** Abdul Rafay, Chaudhary Abdul Fatir, Halaila-Tul Hiba, Manahil Jamil, Muhammad Talha Awan

**Affiliations:** 1 Internal Medicine, Ameer-ud-din Medical College/Lahore General Hospital, Lahore, PAK; 2 Trauma and Orthopaedics, Our Lady's Hospital, Navan, IRL; 3 Ophthalmology, Lahore General Hospital, Lahore, PAK; 4 Internal Medicine, Lahore General Hospital, Lahore, PAK; 5 Psychology, Rawalpindi Medical University, Rawalpindi, PAK; 6 Emergency Medicine, Yeovil District General Hospital, Yeovil, GBR

**Keywords:** post-stroke recovery, stroke, young adult male, ischemic cerebrovascular disease, hyperhomocysteinemia (hhcy)

## Abstract

This case report details the sudden onset of an ischemic stroke in a man in his late 20s, attributed to elevated homocysteine levels. Despite his young age, the patient exhibited increased homocysteine levels, a recognized stroke risk factor. This report underscores the critical importance of recognizing hyperhomocysteinemia as a potential underlying cause of strokes, even in younger age groups. Following ischemic stroke-directed treatment along with the addition of folic acid, vitamin B6, vitamin B12, and methylcobalamin, the patient's condition improved, leading to discharge with normalized homocysteine levels. Highlighting the significance of identifying this risk factor is particularly essential in regions like Pakistan, where a notably high prevalence of hyperhomocysteinemia has been reported. This case serves as a poignant reminder of the need for comprehensive stroke evaluations, urging medical practitioners to consider homocysteine as a potential contributing factor, even when dealing with young and healthy patients.

## Introduction

Stroke stands as the second leading cause of global mortality, emerging as a significant contributor to disability [1]. Particularly pronounced in low- to middle-income countries, the prevalence of stroke surpasses that in high-income counterparts, a divide that continues to widen [2]. Collectively, these lower- and middle-income nations bear the weight of 70% of stroke cases, accounting for a substantial 87% of stroke-related mortalities and disability-adjusted life years [3]. Acquiring a precise understanding of local stroke incidence and predisposing factors is crucial for accurate diagnoses, especially within high-risk demographic subsets.

Hospital-based studies in Pakistan have notably underscored an increased frequency of young stroke patients [4,5]. Existing literature points towards the prevalence of homocysteinemia among young Pakistani individuals suffering from ischemic strokes [6].

Homocysteine, an amino acid with sulfhydryl content, plays a pivotal role as a key intermediate in the metabolism of methionine and cysteine [7]. Its serum levels can elevate in various situations and metabolic disorders, encompassing congenital enzyme deficiencies, chronic renal and hepatic failure, and medication treatment [8]. However, the precise significance of hyperhomocysteinemia as a risk factor for stroke and cardiovascular disease remains a subject of controversy [9].

This report elucidates the case of a 28-year-old man who experienced a sudden ischemic stroke attributed to hyperhomocysteinemia.

## Case presentation

A young man in his 20s sought emergency medical attention at Lahore General Hospital, Pakistan, with a one-day history of left-sided upper and lower limb weakness. The weakness manifested suddenly and exhibited a progressively worsening pattern. Concurrently, the patient reported experiencing mild to moderate headaches intermittently for the past eight hours. Notably, the individual also had a single episode of generalized convulsions accompanied by fecal and urinary incontinence, followed by a post-ictal phase of drowsiness. Absent were concurrent symptoms of fever, nausea, vomiting, photophobia, rash, or joint pains. The patient denied any history of smoking or alcohol consumption. Past medical and surgical history were unremarkable. Detailed family history for any such symptoms, including any atherosclerotic diseases, was not given or documented.

Upon initial examination, the patient's vital signs indicated normal readings, except for an elevated blood pressure reading of 160/100 mmHg and a body mass index (BMI) recorded as 32 kg/m².

The systemic examination, particularly of the central nervous system, revealed a Glasgow Coma Scale (GCS) score of E4V5M6 (15/15), with intact higher mental functions and cranial nerves. Bilateral pupillary reactions to light were observed, and plantar reflexes demonstrated bilateral withdrawal. Deep tendon reflexes were present, and clonus was absent. In addition, there were no signs of meningeal irritation, and muscle strength was 5/5 in the right-sided limbs and 4/5 in the left-sided limbs, particularly in the lower limb as compared to the upper-limb muscle groups. Sensory examination was normal bilaterally. The examination of the rest of the systems yielded unremarkable findings. Table 1 shows the investigations performed when the patient presented in the emergency department.

**Table 1:** Initial investigations performed in the emergency department PT: prothrombin time, INR: international normalized ratio, APTT: activated partial thromboplastin time, ALT: alanine aminotransferase, AST: aspartate aminotransferase

Tests	Results	Reference range
Complete blood count		
Hemoglobin	11.9 gm/dl	13-16.5 g/dl
Total leukocyte count	10.8 *10^03 ^/ul	4-11.0 * 10^03 ^/ul
Platelets	226 *10^03^/ul	10-400 * 10 ^03 ^/ul
Coagulation profile		
PT	12 sec	12-16.5 sec
INR	1.0 sec	0.8-1.2 sec
APTT	28 sec	26-28 sec
Renal function tests (RFTs)		
Serum urea	32 mg/dl	15-40 mg/dl
Serum creatinine	1.1 mg/dl	0.4-1.3 mg/dl
Liver function tests (LFTs)		
Serum total bilirubin	0.8 mg/dl	0.2-1.0 mg/dl
ALT	121 U/L	upto 40 U/L
AST	180 U/L	upto 40 U/L
Alkaline phosphatase	120 U/L	42-306 U/L
Serum electrolytes		
Serum sodium	141 mmol/L	135-155 mmol/L
Serum potassium	4.0 mmol/L	3.5-5.1 mmol/L
Serum chloride	101 mmol/L	98-107 mmol/L
Serum calcium	9 mg/dl	8.5-10.5 mg/dl
Serum magnesium	1.9 mg/dl	1.6-2.5 mg/dl
Random blood sugar	129 mg/dl	120-140 mg/dl

Investigations

All the initial blood tests, including CBC, coagulation profile, renal function tests, and electrolytes, were normal except for raised alanine transaminase (ALT) and aspartate transaminase (AST) levels (Table 1).

Given that stroke secondary to emboli or hemorrhage is the most predominant presentation among patients, a plain head computed tomography (CT) scan was conducted in the emergency setting to rule out both of our primary differentials.

Following the unremarkable findings in the initial emergency evaluation, the young patient was subsequently admitted to the Internal Medicine ward for a comprehensive diagnostic exploration to confirm the cause of the stroke presentation at a young age. Investigations in the ward included an electrocardiogram (ECG) showing a heart rate of 88 beats per minute within a sinus rhythm, an echocardiography revealing a normal study with good biventricular systolic function, and a carotid Doppler reporting bilaterally patent Internal carotid, common carotid, and vertebral arteries with a normal forward flow. In addition, a 24-48-hour Holter monitoring was performed, ruling out the possibility of atrial fibrillation.

**Figure 1:**
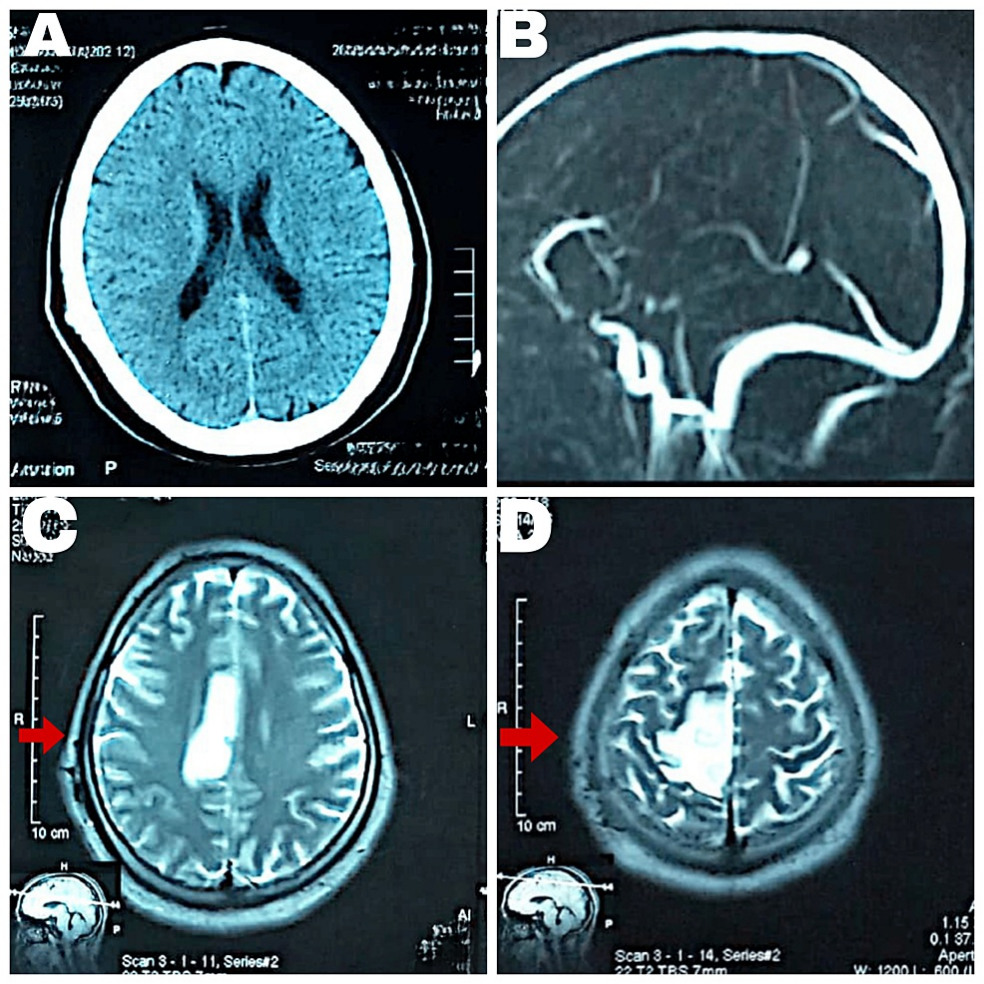
(A & B) Insignificant findings in the CT scan and magnetic resonance venography. (C & D) Ischemic infarct along the anterior cerebral artery in the MRI scan. The arrows show areas damaged by a vascular insult as a bright lesion in FLAIR imaging. FLAIR: fluid-attenuated inversion recovery

CT brain and magnetic resonance venography (MRV) showed insignificant findings (Figure 1A, 1B). However, magnetic resonance imaging (MRI) brain investigations revealed a well-demarcated right-sided top parietal ischemic infarct along the anterior cerebral artery (ACA) territory with a paucity of cortical veins owing to surrounding edema (Figure 1C, 1D).

Further laboratory investigations were ordered and are detailed in Table 2. Notably, all results fell within the normal range except for serum homocysteine levels.

**Table 2:** Lab Investigations during the hospital stay The results depict raised serum homocysteine (fasting) levels. HDL: high-density lipoprotein

Tests	Results	Reference range
Lipid profile		
Serum total cholesterol	146 mg/dl	<200 mg/dl
Triglycerides	33 mg/dl	<150 mg/dl
HDL cholesterol	39 mg/dl	>60 mg/dl
LDL cholesterol	92 mg/dl	60-130 mg/dl
Non-HDL cholesterol	107 mg/dl	<130 mg/dl
Thrombophilia profile		
Protein C	71.4 %	69.1-140%
Free protein S	94.1%	67-140%
Anti-thrombin	118.4%	75.1-125%
Lupus anticoagulant A1	40.6 s	31-44 s
Serum homocysteine (fasting)	27.02 umol/L	5.46-16.2 ymol/L

Differential diagnosis

During the initial emergency evaluation, a CT scan yielded unremarkable results, effectively ruling out the possibility of a hemorrhagic stroke and pointing toward an ischemic stroke as the likely diagnosis. Notably, the patient lacked a smoking history and a positive family history of ischemic strokes, excluding it as a potential cause of the stroke. A lipid profile test confirmed the absence of hypercholesterolemia, and comprehensive cardiac assessments (ECG, ECHO, 24-hour Holter monitoring, and carotid Doppler) detected no cardiac abnormalities, atherosclerotic plaques, or thrombi. Furthermore, normal levels of antiphospholipid A1, protein C, protein S, and anti-thrombin effectively ruled out thrombophilia. However, the patient did exhibit elevated blood pressure (160/100 mmHg) with a raised BMI, which could have contributed to the ischemic stroke. Importantly, fasting serum homocysteine levels were well above the normal limit, suggesting that homocysteine was the possible cause of the ischemic stroke. Furthermore, MRV ruled out venous sinus thrombosis, and MRI confirmed the ACA infarct.

Treatment

After the positive results of the MRI and elevated serum homocysteine levels, the admitted patient was initiated on conservative management involving anti-platelets, antiepileptics, and physiotherapy. After one week, he was discharged with a prescription for folic acid, vitamin B6, vitamin B12, and methylcobalamin. In addition, the patient received standard prophylaxis for ischemic stroke, which included statins and dual anti-platelet therapy. He was counseled regarding physiotherapy, and lifestyle modifications (i.e., exercise, low-salt diet, and diet rich in protein and leafy green vegetables) and scheduled for a follow-up in four weeks to assess serum homocysteine, folate, and vitamin B12 levels.

Outcome and follow-up

In the subsequent four-week follow-up, the patient's condition exhibited substantial improvement, allowing him to resume his daily routine. His follow-up serum homocysteine levels were 5.63 umol/L (normal), serum folic acid (folate) was 15.2 ng/ml (normal), and vitamin B12 was 692 pg/ml (normal).

## Discussion

This report details a distinctive case of ischemic stroke in a young male in his twenties, with elevated serum homocysteine levels. Importantly, hyperhomocysteinemia is recognized as a multifaceted disorder impacting various systems, including the eyes, skin, skeleton, neurological system, and vascular system [10].

In our case, involvement of the cerebral vascular system resulted in stroke-like manifestations primarily affecting the patient's left side. This research highlights that within the cohort of young patients (18-44 years old), ischemic strokes are most commonly attributed to major artery diseases [11]. Other distinctive causes of ischemic stroke in young adults include inherited thrombophilia or acquired hypercoagulable states, such as hyperhomocysteinemia, which was the underlying cause in our patient [12]. Our patient had no other features of hyperhomocysteinemia, such as epilepsy, osteoporosis, hypothyroidism, and gastrointestinal disorders. [13]

Our initial differential diagnoses primarily considered ischemic strokes triggered by embolic phenomena, cerebral venous thrombosis, and brain abscess. There is substantial evidence indicating a correlation between hyperhomocysteinemia and atherothrombotic disorders. For patients with no signs of vascular disease or thrombosis, screening is recommended, particularly in those with an early-onset ischemic stroke and a family history of early atherosclerosis [14].

Existing literature highlights potential mechanisms through which elevated plasma homocysteine levels can lead to toxicity and vascular endothelial damage, where asymmetric dimethylarginine (ADMA) is inhibited by Hcy, reducing the bioavailability of NO by acting as an endothelial nitric oxide synthase (eNOS) inhibitor. [15]. Neurotoxicity and imbalances in other prothrombotic variables can occur as a consequence. Notably, plasma total homocysteine has been identified as a strong, graded, and independent risk factor for coronary heart disease, vascular disease, and stroke, underscoring its clinical significance [16]. Schi et al. indicated that higher homocysteine levels are associated with elevated mortality rates from stroke and coronary heart disease [17].

Normal fasting plasma homocysteine concentrations range from 5 to 15 µmol/L, while hyperhomocysteinemia is categorized as moderate (15-30 µmol/L), intermediate (31-100 µmol/L), or severe (≥100 µmol/L) [18]. Our patient's serum homocysteine levels fell within the moderately raised category at 27.02 µmol/L. The high incidence of folate and vitamin B6 deficiency in Pakistan contributes to the prevalence of hyperhomocysteinemia as deficiency of folate, and vitamin B leads restricts the cofactors necessary for transsulfuration or transmethylation [19].

Stroke syndromes manifest as rapid-onset neurological deficits, with symptoms determined by the affected brain region's vascular anatomy. While certain features lean toward hemorrhagic or ischemic strokes, none decisively distinguish stroke type. In the acute phase, brain and neurovascular imaging are essential. Neurovascular imaging includes CT of the brain, CT angiography, MRI, MRV, and arteriography. Because it is quick and widely available, non-contrast CT of the head is currently the initial investigation of choice [20].

Our patient's unenhanced CT brain conducted shortly after presentation exhibited unremarkable findings. Subsequent MRI disclosed ischemic changes in the parietal lobe within the ACA territory.

## Conclusions

This report underscores the importance of adopting a comprehensive approach to understanding stroke etiologies.

Hyperhomocysteinemia, while relatively infrequent as a contributor to stroke, assumes paramount significance, especially in regions like Pakistan. Recognition within the medical community becomes crucial. Reports highlighting the heightened prevalence of hyperhomocysteinemia among seemingly healthy individuals in Pakistan emphasize the need for increased awareness regarding this specific etiology.

Limited follow-up, lack of comparative case studies, and genetic testing for enzyme mutations, such as methylenetetrahydrofolate reductase (MTHFR), were the limitations in our case.

Furthermore, there is a pertinent call for extensive research to accurately ascertain the prevalence of stroke cases linked to hyperhomocysteinemia. By embracing this holistic perspective and promoting awareness, healthcare professionals can better identify and manage stroke cases attributed to hyperhomocysteinemia, ultimately leading to improved patient outcomes.

## References

[1] Katan M, Luft A (2018). Global burden of stroke. Semin Neurol.

[2] Rahbar MH, Medrano M, Diaz-Garelli F (2022). Younger age of stroke in low-middle income countries is related to healthcare access and quality. Ann Clin Transl Neurol.

[3] Lanas F, Seron P (2021). Facing the stroke burden worldwide. Lancet Glob Health.

[4] Syed NA, Khealani BA, Ali S (2003). Ischemic stroke subtypes in Pakistan: the Aga Khan University Stroke Data Bank. J Pak Med Assoc.

[5] Yusuf Yusuf, S S, Ôunpuu Ôunpuu, S S (2001). Tackling the growing epidemic of cardiovascular disease in South Asia∗☆. J Am Coll Cardiol.

[6] Niazi F, Aslam A, Khattak S, Waheed S (2019). Frequency of homocysteinemia in young ischemic stroke patients and its relationship with the early outcome of a stroke. Cureus.

[7] Zhang T, Jiang Y, Zhang S (2020). The association between homocysteine and ischemic stroke subtypes in Chinese: a meta-analysis. Medicine (Baltimore).

[8] Kumar A, Palfrey HA, Pathak R, Kadowitz PJ, Gettys TW, Murthy SN (2017). The metabolism and significance of homocysteine in nutrition and health. Nutr Metab (Lond).

[9] Chrysant SG, Chrysant GS (2018). The current status of homocysteine as a risk factor for cardiovascular disease: a mini review. Expert Rev Cardiovasc Ther.

[10] Nyhan W, Hoffmann G, Al-Aqeel AI, Barshop B (2003). Homocystinuria. Physician's Guide to the Laboratory Diagnosis of Metabolic Diseases.

[11] Kulesh AA, Nurieva YA, Syromyatnikova LI (2021). Causes of ischemic stroke in patients younger than 45 years: analysis of data from the regional vascular center. Neurology, Neuropsychiatry, Psychosomatics.

[12] George MG (2020). Risk factors for ischemic stroke in younger adults: a focused update. Stroke.

[13] Al Mutairi F (2020). Hyperhomocysteinemia: clinical insights. J Cent Nerv Syst Dis.

[14] Terwecoren A, Steen E, Benoit D, Boon P, Hemelsoet D (2009). Ischemic stroke and hyperhomocysteinemia: truth or myth?. Acta Neurol Belg.

[15] Kwon HM, Lee YS, Bae HJ, Kang DW (2014). Homocysteine as a predictor of early neurological deterioration in acute ischemic stroke. Stroke.

[16] Di Minno MN, Tremoli E, Coppola A, Lupoli R, Di Minno G (2010). Homocysteine and arterial thrombosis: challenge and opportunity. Thromb Haemost.

[17] Shi Z, Guan Y, Huo YR (2015). Elevated total homocysteine levels in acute ischemic stroke are associated with long-term mortality. Stroke.

[18] Ashjazadeh N, Fathi M, Shariat A (2013). Evaluation of homocysteine level as a risk factor among patients with ischemic stroke and its subtypes. Iran J Med Sci.

[19] Iqbal MP (2006). Hyperhomocysteinemia and coronary artery disease in Pakistan. J Pak Med Assoc.

[20] Musuka TD, Wilton SB, Traboulsi M, Hill MD (2015). Diagnosis and management of acute ischemic stroke: speed is critical. CMAJ.

